# Lignin-Derived Diphenylamines for Thermo-Oxidative Stabilization of Natural Rubber

**DOI:** 10.3390/polym18202526

**Published:** 2026-10-15

**Authors:** Zhongwei Wang, Zhaozhuo Yu, Zhu Liu, Qinghan Zeng, Mei Luo, Yipeng Tang, Ning Ji, Wulin Yang, Shoulu Yang

**Affiliations:** 1 Guizhou Academy of Forestry, Guiyang 550005, China; zhongweiwang202204@163.com (Z.W.);; 2 College of Materials Science and Engineering, Central South University of Forestry and Technology, Changsha 410000, China

**Keywords:** lignin, diphenylamine, natural rubber, thermo-oxidative aging

## Abstract

Sustainable polymer stabilization requires renewable antioxidants that combine efficient radical scavenging with clear structure–performance relationships. Diphenylamine antioxidants are widely used in rubber protection, but their conventional production depends on petrochemical feedstocks and hazardous aniline chemistry. Lignin valorization research largely targets model compounds; native lignin’s complexity makes direct conversion challenging. Here, we report a two-step tandem strategy that converts raw lignin into diphenylamine compounds without intermediate purification. PtFe/NiAl_2_O_4_ and phosphotungstic acid first depolymerize lignin selectively to p-substituted phenols, which are then converted into diphenylamine derivatives with ammonium formate, Pd/C and LiOH. The process affords p-substituted phenols in 35 wt% yield and final diphenylamine compounds in 23 wt% yield from birch lignin. When incorporated into natural rubber at 1 wt%, the lignin-derived diphenylamines suppress thermo-oxidative aging: after treatment at 140 °C for 24 h, the mass loss decreases from 10% for the untreated rubber to 1% for the stabilized material. The stabilized rubber also withstands harsher aging conditions and shows performance comparable to commercial EPDM rubber. Mechanistic experiments indicate that lignin demethoxylation supplies in situ hydrogen for depolymerization, while the diphenylamine N–H group interrupts radical chain oxidation in the rubber matrix. This work links lignin valorization chemistry with polymer-aging control and provides a renewable route to high-performance rubber antioxidants.

## 1. Introduction

Natural rubber is used in tires, seals and many other industrial and consumer products because of its elasticity and mechanical resilience [1,2]. However, the unsaturated polymer backbone is readily damaged by thermo-oxidative aging, in which heat, oxygen and light initiate radical chain reactions [3]. This degradation reduces mechanical integrity and shortens service life [4]. High-efficiency antioxidants are therefore essential for rubber stabilization. Among the available stabilizers, amine antioxidants, especially diphenylamine and its derivatives, are valued for their ability to quench free radicals more efficiently than many phenolic alternatives [5].

The sustainability of these stabilizers remains a concern. Commercial diphenylamines are commonly prepared from non-renewable petrochemical feedstocks, and routes based on aniline–phenol or cyclohexylamine condensation are energy-intensive [6]. They also consume large amounts of aniline, a hazardous reagent that raises safety and health concerns [7,8]. Developing a renewable and safer route to diphenylamine antioxidants would therefore benefit both polymer stabilization and the rubber additive industry.

Lignin is the most abundant renewable aromatic polymer on Earth, accounting for 15–35% of lignocellulosic biomass [9], with annual isolated production of roughly 50 million metric tons [10]. Its guaiacyl (G), p-hydroxyphenyl (H) and syringyl (S) units [11,12] provide a potential source of phenols and aromatic amines [13,14]. Previous work on C–O/C-C bond cleavage and C–N bond formation has often relied on low-molecular-weight lignin models, especially β-O-4 dimers. For example, β-O-4 model compounds have been converted into benzylamines through C α-OH dehydrogenation and Cβ-O bond hydrogenolysis [15]. Oxidized β-O-4 models have also undergone copper-catalyzed amine-mediated oxidative cleavage to form aromatic amides [12], and KOH-promoted amine-involved [4 + 2] cyclization has produced biologically active 2,6-diphenylpyridines [16]. Dieter et al. employed a Cu-ZrO_2_-catalyzed borrowing hydrogen method to synthesize tertiary amines from the lignin model monomer 3-(3,4-dimethoxyphenyl)-1-propanol and the lignin-derived practical monomers, 3-(4-hydroxyphenyl)-1-propanol and dihydroconiferyl alcohol, using dimethylamine as the nitrogen source [17]. As another example, renewable homopolymers and block copolymers (BCPs) were synthesized via functionalization of the lignin-derived vanillin and reversible addition–fragmentation chain transfer (RAFT) polymerization [18]. These reactions establish useful chemistry, but translating model compound reactivity to the heterogeneous structure of real lignin remains challenging.

To address this gap, we developed a two-step tandem strategy that converts raw lignin directly into diphenylamine compounds. In the first step, lignin is directionally depolymerized to p-substituted alkylphenols, which are suitable intermediates for subsequent C–N bond formation. In the second step, these phenolic intermediates undergo amination condensation using ammonium formate as the nitrogen source and palladium on carbon as the reducing catalyst.

The amination step depends strongly on the selectivity of lignin depolymerization. Pt/NiAl_2_O_4_ has been reported to depolymerize lignin selectively into para-alkylphenols without external hydrogen [19], and bimetallic PtNi catalysts further improve the yield of p-substituted phenols [20]. Proton-assisted reductive demethoxylation can also promote the formation of p-substituted phenols [21]. On this basis, we combined a bimetallic PtFe/NiAl_2_O_4_ catalyst with protonic acid activation to improve the production of p-substituted phenols from raw lignin.

A further obstacle in lignin valorization is the cost and solvent demand associated with separating complex depolymerization mixtures [22]. Our process avoids this barrier by using a simple extraction after depolymerization and feeding the crude phenolic fraction directly into the amination step. This study establishes a direct, low-carbon route from raw lignin to high-value rubber antioxidants without intermediate separation. The two-step tandem process first depolymerizes lignin over PtFe/NiAl_2_O_4_ and phosphotungstic acid, using in situ H_2_ (generated via demethoxylation) to afford 35 wt% p-alkylphenols. These intermediates are then aminated with ammonium formate over Pd/C and LiOH, yielding 23 wt% diphenylamine derivatives. When incorporated into natural rubber at 1 wt%, these derivatives suppress radical chain oxidation through N–H hydrogen donation. Consequently, the mass loss during accelerated thermal aging (140 °C, 24 h) drops from 10% to 1%, and the antioxidant performance is comparable to that of commercial EPDM and DDA. This study shows that diphenylamine compounds, when used directly as antioxidants in natural rubber, deliver antioxidant performance comparable to that of commercial EPDM rubber. This strategy provides a new pathway for developing sustainable, high-performance, and low-cost antioxidant technologies for natural rubber (Figure 1).

## 2. Materials and Methods

### 2.1. Materials

Birch, poplar, camphor and beech powders (200 mesh) were purchased from Guizhou Kaifeng Chemical & Glass Co., Ltd. (Guiyang, China). Iron nitrate (AR 98%), aluminum nitrate (AR, 99%), chloroplatinic acid hydrate (AR, 99.9%), phosphotungstic acid hydrate (AR), ammonium formate (AR, 99%), palladium on activated charcoal (1 wt% loading, dry basis), lithium hydroxide (AR, 99%), magnesium oxide (AR, 98%), potassium hydroxide (AR, 95%) and calcium oxide (AR, 98%) were used as received. The preparation of antioxidant rubber was supported by Guizhou Dazhong Rubber Co., Ltd. (Guiyang, China), which provided the experimental site and processing environment.

### 2.2. Preparation of Catalyst

NiAl_2_O_4_ spinel was synthesized by co-precipitation with a Ni/Al molar ratio of 1:2. Typically, 40 mmol of Ni(NO_3_)_2_·6H_2_O and 80 mmol of Al(NO_3_)_3_·9H_2_O (using anhydrous salts) were dissolved in 200 mL of ultrapure water (18.2 MΩ·cm) under stirring at room temperature. The pH was adjusted to 8 by dropwise addition of ammonia solution. After 1 h of stirring, the precipitate was aged overnight, filtered, and rinsed with deionized water until the filtrate was neutral (pH 7). The solid was dried at 100 °C for 12 h and calcined at 800 °C under N_2_ for 8 h. PtFe/NiAl_2_O_4_ was then obtained via incipient wetness impregnation with 10% H_2_PtCl_6_ and 10% Fe(NO_3_)_3_ aqueous solutions. The impregnated sample was dried at 80 °C overnight, calcined at 450 °C for 4 h under N_2_ (heating rate: 5 °C min^−1^), and finally reduced at 350 °C under 10% H_2_/Ar (same heating rate) before use (Figure S1).

### 2.3. Characterization

Structural characterization was performed using X-ray diffraction (XRD), transmission electron microscopy (TEM), and scanning transmission electron microscopy (STEM). XRD patterns were recorded in θ–2θ mode on a D8 focusing diffractometer (Bruker, Billerica, MA, USA) with Cu Kα_1_ radiation (λ = 1.5406 Å), operating at 40 kV and 40 mA, over a 2θ range of 10–80°. TEM images were acquired on a JEOL 2100F microscope (JEOL, Tokyo, Japan) at 200 kV. STEM and high-angle annular dark-field (HAADF-STEM) imaging were carried out on a ThermoFisher Talos F200X microscope (ThermoFisher, Waltham, MA, USA), using a convergence semi-angle of 11 mrad and inner/outer collection angles of 59/200 mrad. Redox properties were evaluated by H_2_ temperature-programmed reduction (H_2_-TPR) on a Huasi DAS-7200 automatic chemisorption apparatus (Hunan Huasi Instruments Co., Ltd., Changsha, China). Approximately 0.1 g of catalyst, loaded in a quartz tube, was first degassed and dehydrated, then heated from 20 to 800 °C at a ramp rate of 10 °C min^−1^. Surface composition was analyzed by X-ray photoelectron spectroscopy (XPS) on a Thermo Scientific Escalab 250Xi instrument (ThermoFisher, Waltham, MA, USA) with monochromatic Al Kα radiation. Textural properties were determined from N_2_ adsorption–desorption isotherms measured on a Micromeritics ASAP 2020 M analyzer (Micromeritics, Norcross, GA, USA). Prior to measurement, samples were degassed at 200 °C for 6 h, and specific surface areas were calculated using the Brunauer–Emmett–Teller (BET) method. The molecular weight of lignin before and after the reaction was measured by gel permeation chromatography (GPC), giving the number-average molecular weight (M_n_). Additionally, rubber aging tests were conducted according to the Chinese standard GB/T 3512-2014 [23].

### 2.4. Selective Conversion of Lignin to P-Substituted Monophenols

Reaction procedure. Lignin depolymerization was carried out in a 50 mL PTFE-lined stainless-steel autoclave. In a typical run, 0.10 g of lignin, 0.20 g of PtFe/NiAl_2_O_4_ catalyst, 15.0 mL of ultrapure water, and 0.10 g of phosphotungstic acid (HPW) were charged into the reactor (calibrated volume: 50.4 mL). The mixture was stirred at 600 rpm throughout the reaction. The autoclave was purged three times with high-purity N_2_ (99.999%) and then pressurized to 2 MPa N_2_. Subsequently, the reactor was rapidly heated to the target temperature and maintained under vigorous stirring for the desired reaction time.

Product workup. After the reaction, the autoclave was immediately cooled in an ice-water bath. The liquid phase was extracted with 5 mL of ethyl acetate, and the resulting mixture was separated by centrifugation at 12,000 rpm. Analysis. Product identification was performed by GC-MS (Agilent 7890B-5975C, Agilent, Santa Clara, CA, USA), and quantification was carried out by GC-FID using external standards. The total molar yield of p-substituted phenols derived from lignin was calculated as follows:

p-Substituted phenols (mol%) = (moles of phenol + p-cresol + p-ethylphenol + p-propylphenol)/(total moles of lignin-derived monomers) × 100%

The total monomer content of each lignin feedstock is summarized in Table S1.

### 2.5. Amination Polycondensation of P-Substituted Phenols

The ethyl acetate extract (5 mL) of the lignin depolymerization mixture, containing p-substituted phenols, was transferred to a 25 mL three-way pressure tube and dried under nitrogen. After solvent exchange from water to toluene, the tube was charged with ammonium formate (1 equiv), Pd/C (20 mol%), LiOH (40 mol%), and toluene (0.5 mL), with the p-substituted phenols serving as the 10 equiv substrate. The calibrated reaction volume was 1.2 mL, and the mixture was stirred at 300 rpm. The tube was purged three times with argon, heated to the desired temperature, and held under stirring. After cooling to room temperature, the liquid phase was extracted with 5 mL of acetone and centrifuged at 12,000 rpm. Products were identified by GC-MS and quantified by HPLC-UV with external standards. The molar yield of diphenylamine compounds was expressed relative to the total lignin-derived monomers:

Diphenylamine yield (mol%) = (detected diphenylamines)/(total monomers) × 100%(1)

### 2.6. In Situ Reaction Monitoring of Lignin Model Compounds

In situ monitoring was performed via pyrolysis gas chromatography–mass spectrometry (Py-GC-MS), using 1-(4-methoxyphenyl)ethanol as the lignin model compound under identical reaction conditions. Py-GC-MS measurements were conducted on a Frontier single-shot pyrolyzer (Frontier Laboratories, Koriyama, Japan) coupled to a GCMS-QP 2010 system. The pyrolysis products were separated and identified at 300 °C under a nitrogen atmosphere.

### 2.7. Preparation of Antioxidant Rubber

Diphenylamine-containing natural rubber specimens were prepared following the standard steps of mastication, mixing, molding, vulcanization, and trimming. Briefly, 1000 g of natural rubber was masticated in an internal mixer to soften the chains and improve processability. Then, 1 phr of the diphenylamine antioxidant was added along with conventional compounding agents (zinc oxide, stearic acid, carbon black, and sulfur), and the mixture was thoroughly blended. The compound was sheeted, molded into test blocks under pressure, and vulcanized on a plate press. After curing, the blocks were trimmed and used as test specimens.

### 2.8. Thermal Aging Test

According to GB/T 3512-2014, vulcanized rubber specimens were placed in a hot-air aging oven and aged at the specified temperature and time. After aging, the specimens were removed and tested for mechanical properties.

## 3. Results and Discussion

### 3.1. Properties of Catalyst

The structure and composition of the optimized PtFe/NiAl_2_O_4_ catalyst were examined using complementary characterization methods. Inductively coupled plasma optical emission spectrometry (ICP-OES) confirmed successful metal loading, with Pt and Fe contents of approximately 2.1 wt% and 1.8 wt%, respectively. The morphology, phase structure and surface chemistry were further evaluated by XRD, TEM, HAADF-STEM, energy-dispersive X-ray spectroscopy (EDS), XPS and H_2_-TPR.

The XRD pattern (Figure 2a) shows diffraction peaks at 19.1°, 31.3°, 36.5°, 43.7°, 57.0°, 59.0° and 65.1°, which correspond to the crystalline planes of the NiAl_2_O_4_ support [24] (Figure S2). Additional peaks at 39.8°, 45.2° and 68.0° match the (111), (200) and (220) planes of Pt, whereas peaks at 44.7°, 68.0° and 81.0° correspond to the (110), (200) and (211) planes of Fe [25]. HAADF-STEM and EDS mapping (Figure 2d,e) show that Pt and Fe are uniformly dispersed, with an average particle size of 2.1 nm (Figure S3). Aberration-corrected HAADF-STEM (Figure 2f) reveals lattice spacings of 0.16–0.21 nm, between the standard spacings of Pt (111) (0.202 nm) and Fe (002) (0.166 nm) [26]. Together with the SAED patterns, these measurements support the formation of PtFe alloy nanoparticles [27]. TEM and N_2_ adsorption–desorption analyses (Figure S4) further show a mesoporous structure with a specific surface area of 556.1 m^2^ g^−1^ and a pore volume of 1.43 cm^3^ g^−1^, which should facilitate the adsorption and transport of hydrogen and organic substrates during catalytic reduction.

H_2_-TPR was used to evaluate reducibility and metal–support interactions (Figure 2c). PtFe/NiAl_2_O_4_ shows a distinct H_2_ consumption peak near 208 °C, assigned to co-reduction of Pt and Fe species [28]. The main reduction signal for iron shifts from above 1100 °C to approximately 450 °C, indicating that Pt promotes hydrogen activation and facilitates the reduction of neighboring Fe oxides [29]. XPS analysis further confirms the surface elemental composition and electronic states. The survey spectrum (Figure S5) shows C, N, O, Al, Pt and Fe, and the high-resolution Pt spectrum (Figure 2b) gives a Pt binding energy of 71.4 eV, slightly higher than that of metallic Pt (71.0 eV). This positive shift suggests partial electron transfer from Fe to Pt and further supports PtFe alloy formation [30].

### 3.2. Synthesis of Diphenylamine from Lignin

Diphenylamine synthesis proceeded through a two-step tandem sequence. In the first step (Figure 3a), PtFe/NiAl_2_O_4_ selectively depolymerized lignin into p-substituted phenols. The system did not require external hydrogen because hydrogen was generated in situ from demethylation and aqueous reforming. Acidic protons pre-activated ether bonds, lowering the C–O bond dissociation energy and promoting targeted depolymerization. In the second step (Figure 4a), Pd/C and ammonium formate promoted amination of the p-substituted phenol intermediates to give diphenylamine compounds. The two steps were connected without intermediate purification.

The depolymerization step was first evaluated using different organosolv lignins in the combined bimetallic catalyst and protonic acid system (Figure 3b). Pre-activation of lignin is important for targeted monomer release [31]. Reactions under acidic, alkaline and neutral conditions (Figure 3c) showed that acid-mediated protonation weakened ether linkages and improved monomer formation. Oxygen vacancies on the catalyst supported and active PtFe alloy sites then promoted cleavage of the activated C–O bonds and hydrodeoxygenation [32]. Accordingly, acidic aqueous conditions produced more monomeric phenols than basic or neutral media. However, the acid concentration required careful control: insufficient acid did not activate the ether bonds effectively, whereas excess acid favored benzyl carbocation stabilization, condensation, rearrangement, polymerization and catalyst coking [33].

The reaction temperature, pressure, time, catalyst loading and substrate concentration were optimized using birch lignin as the model substrate (Figure 3d–g and Figure S6). These variables affected the yield of p-alkylphenols, including phenol, p-cresol, p-ethylphenol and p-propylphenol. An excessive temperature, long reaction time or high pressure decreased the product yield by promoting secondary condensation of reactive intermediates [34]. The optimized conditions were 0.2 g of catalyst and 0.1 g of lignin at 280 °C for 20 h under 2 MPa N_2_. Under these conditions, birch lignin gave a total mass yield of 45%, including 35% p-alkylphenols and approximately 10% other aromatics and cycloalkanes. The total molar yield of para-alkylphenols from birch lignin approached 71%.

GPC analysis before and after the reaction (Figure S7) confirmed extensive lignin fragmentation: the weight-average molecular weight decreased from 2345 to 278 in the reaction residue. Catalyst stability was then assessed over five consecutive runs. The total product mass yield decreased moderately from 35% to 25% (Figure S8). Characterization of the spent catalyst (Figure S9 and Table S1) showed a decrease in the BET surface area after the fourth run, whereas the Pt and Fe nanoparticle sizes remained largely stable (Figure S10). Substrate scope experiments with other lignin sources (Table S2) gave similar product distributions, with para-alkylphenol mass and molar yields remaining above 24% and 69%, respectively (Figure 3b). The higher performance of birch lignin is consistent with its higher β-O-4 linkage and syringyl unit contents and its lower proportion of recalcitrant phenolic hydroxyl structures [35].

In the second step, the p-substituted phenols obtained from birch lignin depolymerization were used directly as substrates for diphenylamine synthesis with ammonium formate as the nitrogen source. Catalyst screening (Figure 4b) showed that Pd/C alone gave a low product yield. Removing in situ-generated water was expected to shift the equilibrium, but basic desiccants such as MgO and CaO provided little improvement [36]. Base promoter screening revealed a strong cation effect: KOH almost suppressed amination, whereas LiOH increased the mass yield to 23% based on the initial lignin weight. This difference may arise from the high charge density and coordinating ability of Li^+^, which can bind oxygen-containing intermediates, stabilize transition states and direct condensation toward diphenylamine formation [37,38].

Solvent selection also controlled the amination pathway (Figure 4d). Polar protic solvents, including methanol and ethanol, disrupted diphenylamine formation, probably because hydrogen bonding and proton transfer quenched reactive intermediates and promoted side reactions [39]. Non-polar aprotic solvents were more suitable. Toluene outperformed n-hexane, which may reflect the stabilization of transition states through favorable π interactions [40]. Toluene also performed better than p-xylene under these conditions, possibly because its π-electron cloud is more polarizable in this amination environment [41].

After establishing the catalyst and solvent system, the reaction time, temperature, catalyst loading and component ratio were optimized using lignin-derived p-substituted phenols (Figure 4e–g and Figure S11). Excessively harsh conditions decreased the yield, consistent with secondary thermal degradation of diphenylamines and competing side reactions [42]. The optimized amination conditions were 20 mol% Pd/C, 0.2 mmol LiOH and 10 equiv of p-substituted phenols at 140 °C for 24 h. Under these conditions, the tandem process produced diphenylamine compounds (Figure S12) in 23 wt% yield from raw birch lignin (Figure 4c), and the molar yield from p-substituted phenols was approximately 84%. These results show that genuine polymeric biomass can be converted into functional diphenylamine antioxidants without isolating the phenolic intermediates.

### 3.3. The Pathway for the Conversion of Lignin to Diphenylamine

The depolymerization pathway from birch lignin to p-substituted phenols was examined by time-resolved 2D-HSQC NMR spectroscopy. The spectra (Figure S13) show the rapid attenuation and eventual disappearance of lignin side-chain signals. Signals from methoxy groups (Ar-OCH_3_) also decreased, although they remained detectable at a lower intensity. At the same time, the signals assigned to syringyl (S) and guaiacyl (G) units declined, whereas the p-hydroxyphenyl (H) signal increased. This evolution indicates that lignin first fragments into monomeric species and then undergoes targeted demethoxylation [43].

The depolymerization sequence was further investigated by temperature-programmed surface reaction (TPSR) using dimeric and monomeric model compounds. The β-O-4 model compound α-[(2-methoxyphenoxy)methyl]benzyl alcohol (BM) was used to probe ether linkage cleavage. As shown in Figure 5a, acetophenone and guaiacol formed rapidly at the beginning of the reaction. Guaiacol was subsequently converted into phenol through demethoxylation, confirming a pathway involving ether bond cleavage followed by methoxy removal [44].

The transformation of downstream phenolic intermediates was traced with 4-(3-hydroxypropyl)-2-methoxyphenol (PG-OH), a lignin-derived monomer containing both hydroxyl and methoxy groups. As the reaction proceeded (Figure 5b), ethyl guaiacol formed as the main intermediate, together with smaller amounts of p-substituted phenols, including p-cresol, p-ethylphenol and p-propylphenol. The yield of 4-ethylphenol increased as 4-ethylguaiacol was consumed, indicating that 4-ethylphenol is generated directly by demethoxylation of 4-ethylguaiacol.

Py-GC-MS analysis of 1-(4-methoxyphenyl)ethanol (MP-ol) was used to verify demethoxylation and its contribution to the reductive environment. Under optimal N_2_-purged conditions, the uncatalyzed control did not produce peaks associated with methanol elimination from MP-ol (Figure 5c). In contrast, the PtFe/NiAl_2_O_4_ catalyst generated the characteristic demethanolated intermediate. This observation indicates that catalytic cleavage of lignin methoxy groups produces methanol. The liberated methanol can then undergo aqueous reforming or cracking to generate in situ hydrogen, creating a self-sustaining reductive environment for hydrodeoxygenation [45].

Combining these observations gives the depolymerization pathway shown in Figure 5d,e. p-Cresol and related methyl-substituted products mainly arise from retro-aldol cleavage of β-O-4 linkages. 4-Ethylphenol and related ethyl-substituted products originate from the rearrangement of hydroxypropyl side chains, whereas 4-propylphenol and related propyl-substituted products form through subsequent hydrogenolysis. Ongoing demethylation generates methanol, which acts as an internal hydrogen donor after cracking and promotes hydrogenolysis. These results provide a mechanistic basis for the targeted production of p-substituted phenols from raw lignin and are broadly consistent with previously reported pathways [46].

In this system, phosphotungstic acid protonates the ether oxygen and thereby lowers the activation barrier for C–O bond cleavage, enabling the pre-activation of the ether bonds. Concurrently, the PtFe/NiAl_2_O_4_ catalyst produces active hydrogen in situ through demethoxylation and methanol cracking, which selectively hydrodeoxygenates the pre-activated intermediates without external H_2_. The close spatial proximity of acid and metal sites, combined with the reaction-directing role of the protonic acid, ensures high efficiency and selectivity in the conversion of lignin to alkylphenols, while eliminating the need for external high-pressure hydrogen and reflecting a sustainable, low-carbon approach.

The amination pathway was probed by replacing phenol with cyclohexenone in the amination system. Under standard conditions (140 °C for 18 h; Figure 6b), cyclohexenone produced the proposed intermediate 2a together with the final diphenylamine product. This control experiment supports the role of cyclohexenone as a reactive intermediate that condenses with ammonia to form the dimeric framework.

On the basis of these experiments, we propose the amination pathway in Figure 6c. Ammonium formate first decomposes on Pd(0), generating reactive Pd(II)-dihydride species and releasing carbon dioxide and ammonia. The phenolic substrate is locally reduced by Pd(II)-dihydride to form the corresponding cyclohexenone. Cyclohexenone then condenses with in situ-generated ammonia to give an imine, which undergoes secondary condensation through enamine tautomerization to build the coupled intermediate. Finally, dehydrogenation on Pd(0) restores aromaticity and yields the diphenylamine compound [47].

### 3.4. Antioxidant Performance of Diphenylamine

The biomass-derived diphenylamine compounds were incorporated into natural rubber at 1 wt% to evaluate their stabilizing effect (Figure 7a). The composites were then subjected to accelerated thermal aging. After aging at 140 °C for 24 h (Figure 7b,c), untreated natural rubber showed severe surface cracking and a mass loss of 10%. In contrast, the diphenylamine-stabilized natural rubber retained its surface integrity without visible cracking, and its mass loss was limited to 1%.

The stabilized rubber was further tested under harsher thermal conditions. At 160 °C, the treated rubber still resisted cracking, whereas the untreated rubber failed (Figure S14). At 180 °C for 16 h, the 1 wt% treated sample eventually cracked and showed a mass loss of 5%. Under the same conditions, commercial EPDM rubber also cracked and showed a slightly higher mass loss of 6%. In addition, after aging at 80 °C for 36 h, the tensile strength of the neat rubber decreased markedly (from 24 N to 14 N). In contrast, the rubbers containing the diphenylamine compound or commercial DDA exhibited only minimal changes (from 33 N to 36 N, and from 32 N to 35 N, respectively), indicating comparable antioxidant protection.

These results indicate that the lignin-derived diphenylamine exhibits antioxidant performance comparable to that of the commercial antioxidant DDA. Both DSC and FT-IR are used to evaluate the antioxidant performance of the rubber samples (Figure S15). The oxidation peak of the rubber with added antioxidant shifts to higher temperatures compared to that of neat natural rubber in DSC [48]. In the infrared spectra, the carbonyl absorbance of natural rubber is significantly higher than that of the antioxidant-containing sample [49]. These data consistently demonstrate that the antioxidant developed in this work significantly enhances the thermo-oxidative aging resistance of natural rubber.

The stabilizing effect is consistent with the radical scavenging mechanism of diphenylamine antioxidants (Figure 7d). The diphenylamine derivatives in this system quench peroxyl radicals via N–H hydrogen donation, thus interrupting the radical chain propagation [50]. Meanwhile, the generated nitrogen-centered radicals undergo efficient stabilization by conjugated delocalization over the two phenyl rings, avoiding further chain initiation [51]. This combined quenching and stabilization synergy is responsible for the outstanding thermo-oxidative protection observed in natural rubber [52].

## 4. Conclusions

Diphenylamine antioxidants (e.g., DDA, ODA) are among the most widely used antioxidant classes in the rubber industry. This study establishes a renewable tandem route for producing functionalized diphenylamine antioxidants directly from lignin. PtFe/NiAl_2_O_4_ combined with protonic acid selectively depolymerizes raw lignin to p-substituted phenols, giving a 35 wt% phenolic fraction. Subsequent amination with ammonium formate over Pd/C and LiOH converts these intermediates into diphenylamine compounds in 23 wt% yield from birch lignin, without intermediate separation or purification. When incorporated into natural rubber at 1 wt%, the lignin-derived diphenylamines substantially reduce thermo-oxidative mass loss and delay visible cracking, with performance comparable to commercial EPDM rubber under severe aging conditions. Additionally, its antioxidant performance is equivalent to that of commercial DDA. This work is the first to achieve the direct synthesis of diphenylamine antioxidants from lignin, providing the rubber industry with an alternative route that is independent of petroleum-based feedstocks. This helps to alleviate dependence on fossil resources and reduce the carbon footprint. By connecting lignin depolymerization chemistry, diphenylamine formation and radical-mediated rubber stabilization, this work provides a sustainable route to high-performance polymer additives and clarifies how biomass-derived aromatics can be used to control polymer aging. However, the diphenylamine antioxidant obtained here is a mixture of mono- and di-alkylated derivatives, not a single compound. While its overall performance matches commercial DDA, the individual contributions and synergies remain unclear, hindering accurate quantification of structure–activity relationships.

## Figures and Tables

**Figure 1:**
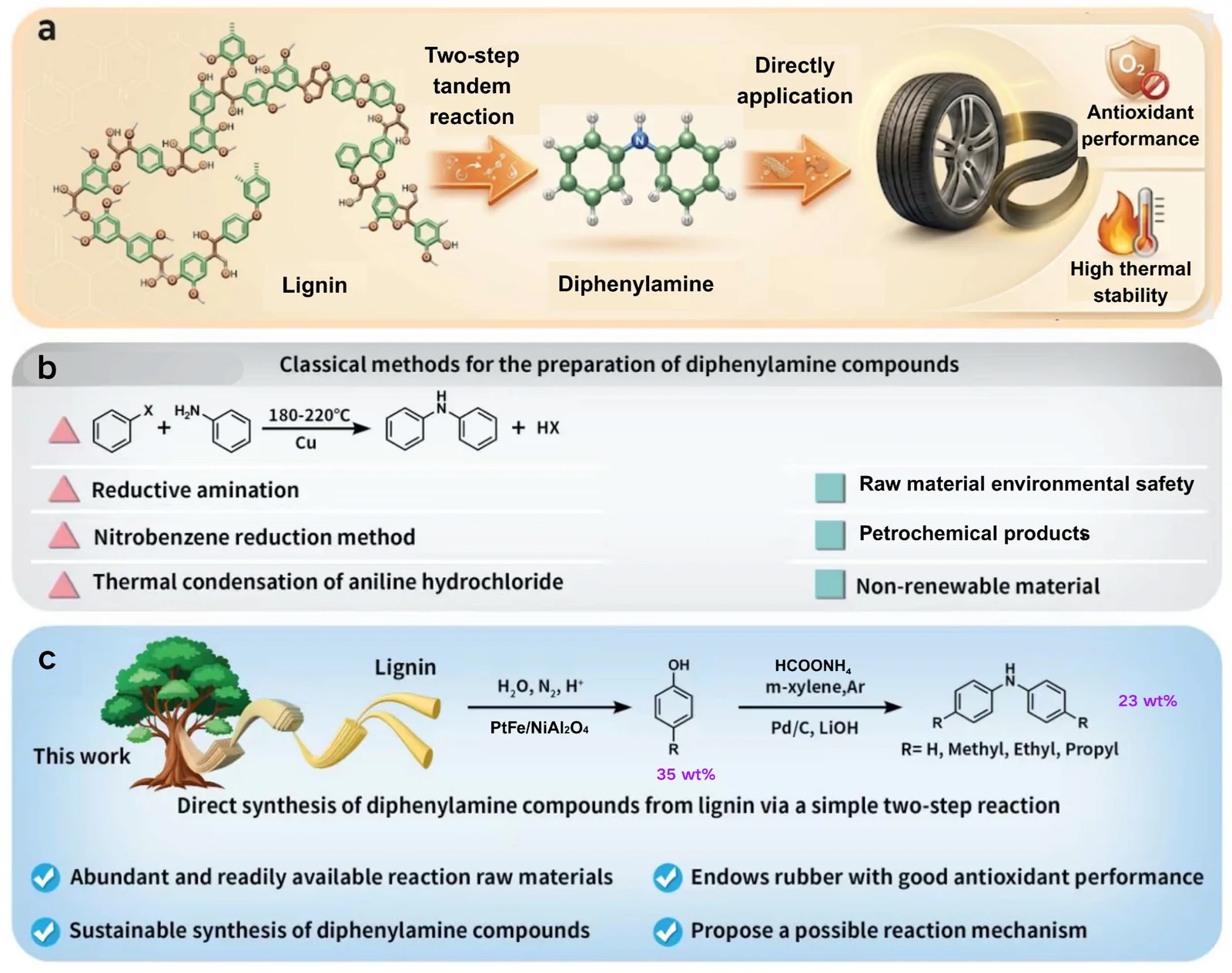
(**a**) Schematic illustration of lignin-to-diphenylamine conversion for enhanced antioxidant performance in rubber. (**b**) Conventional routes to diphenylamine compounds. (**c**) Sustainable synthesis of diphenylamine compounds from lignin.

**Figure 2:**
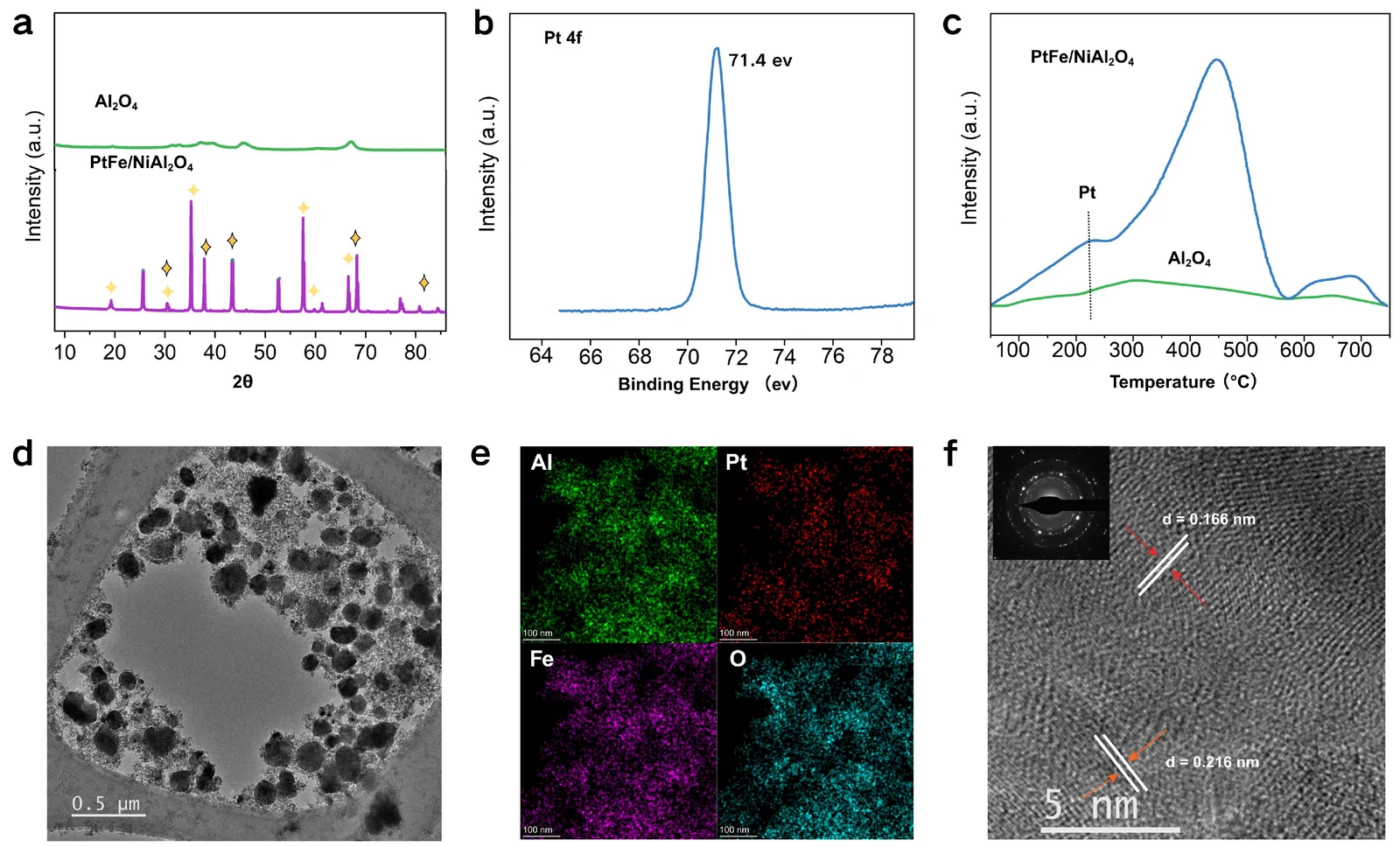
Characterization of the catalyst. (**a**) XRD pattern. (**b**) XPS spectra. (**c**) H_2_-TPR profile. (**d**) TEM images. (**e**) HAADF-STEM image and elemental maps showing the distribution of Al, Pt, Fe and O. (**f**) HRTEM images and SAED pattern.

**Figure 3:**
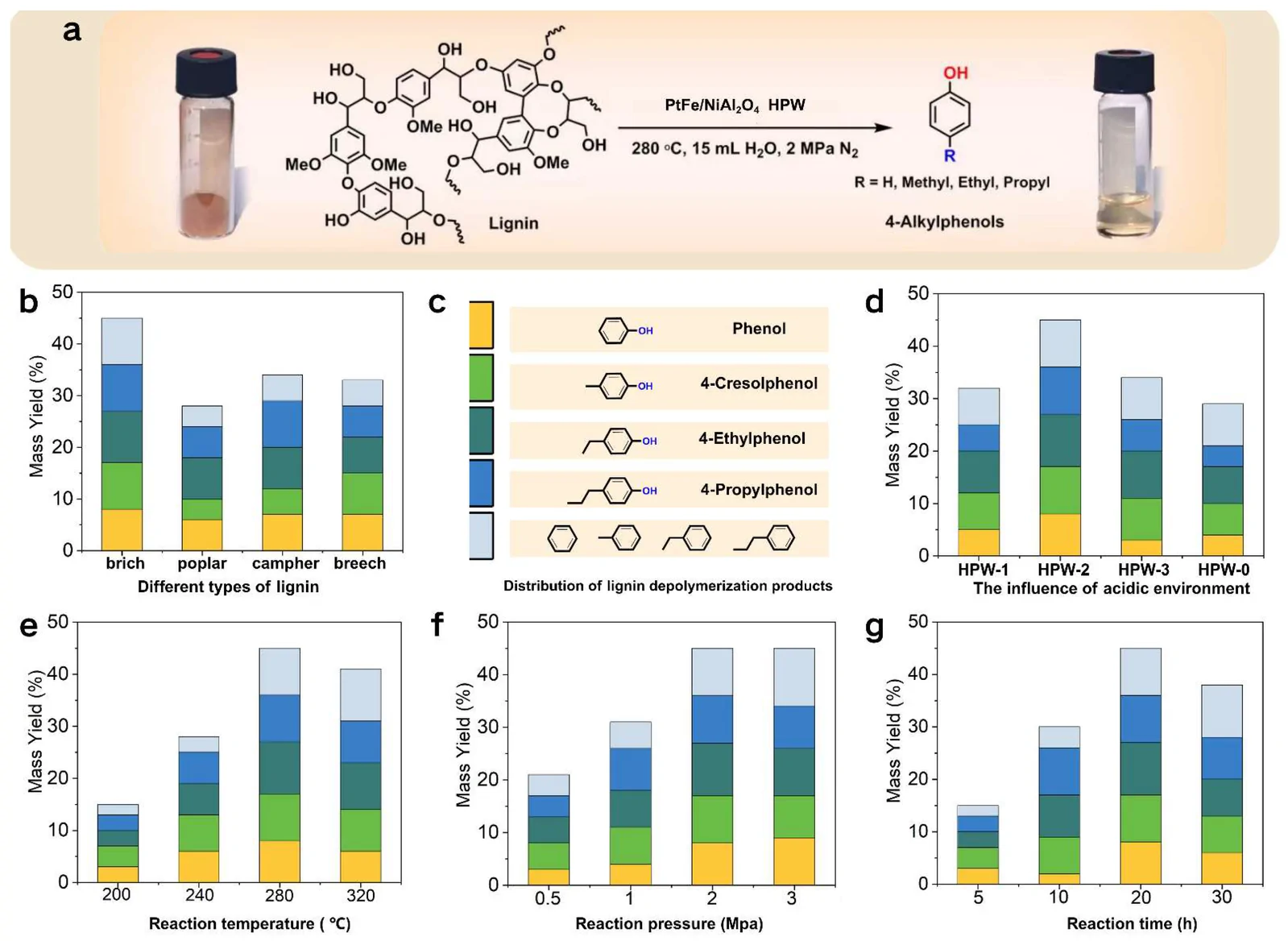
Depolymerization of lignin to p-substituted phenols. (**a**) Reaction scheme. (**b**) Lignins from different wood sources. (**c**) Product distribution after depolymerization. (**d**–**g**) Effects of acidity/basicity, temperature, pressure and reaction time. HPW-1, 2 and 3 correspond to 0.05, 0.10 and 0.20 g of HPW, respectively.

**Figure 4:**
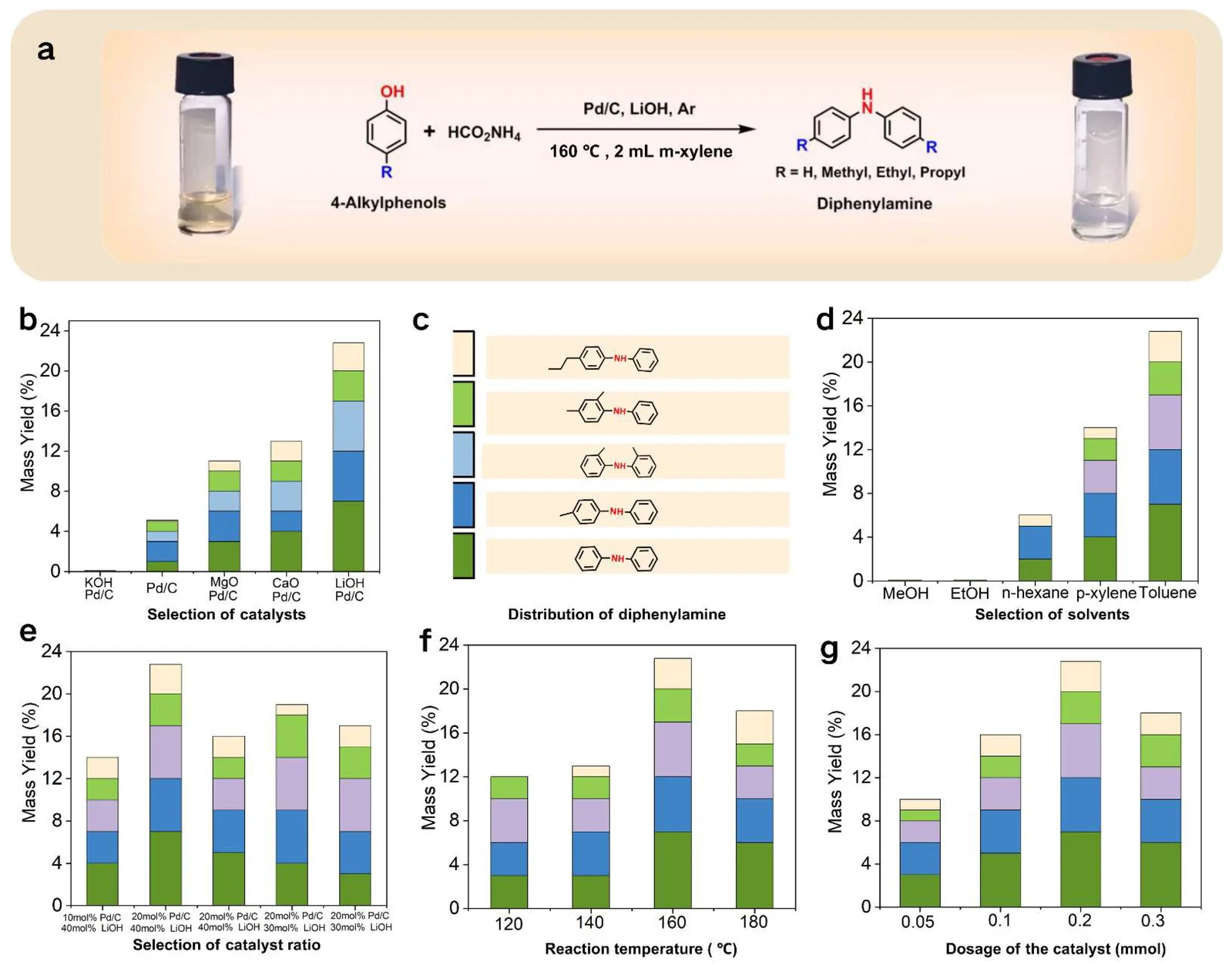
Conversion of p-substituted phenols into diphenylamine compounds. (**a**) Reaction scheme. (**b**) Catalyst screening and adaptation. (**c**) Product distribution. (**d**–**g**) Effects of solvent, catalyst adaptation, temperature and catalyst dosage.

**Figure 5:**
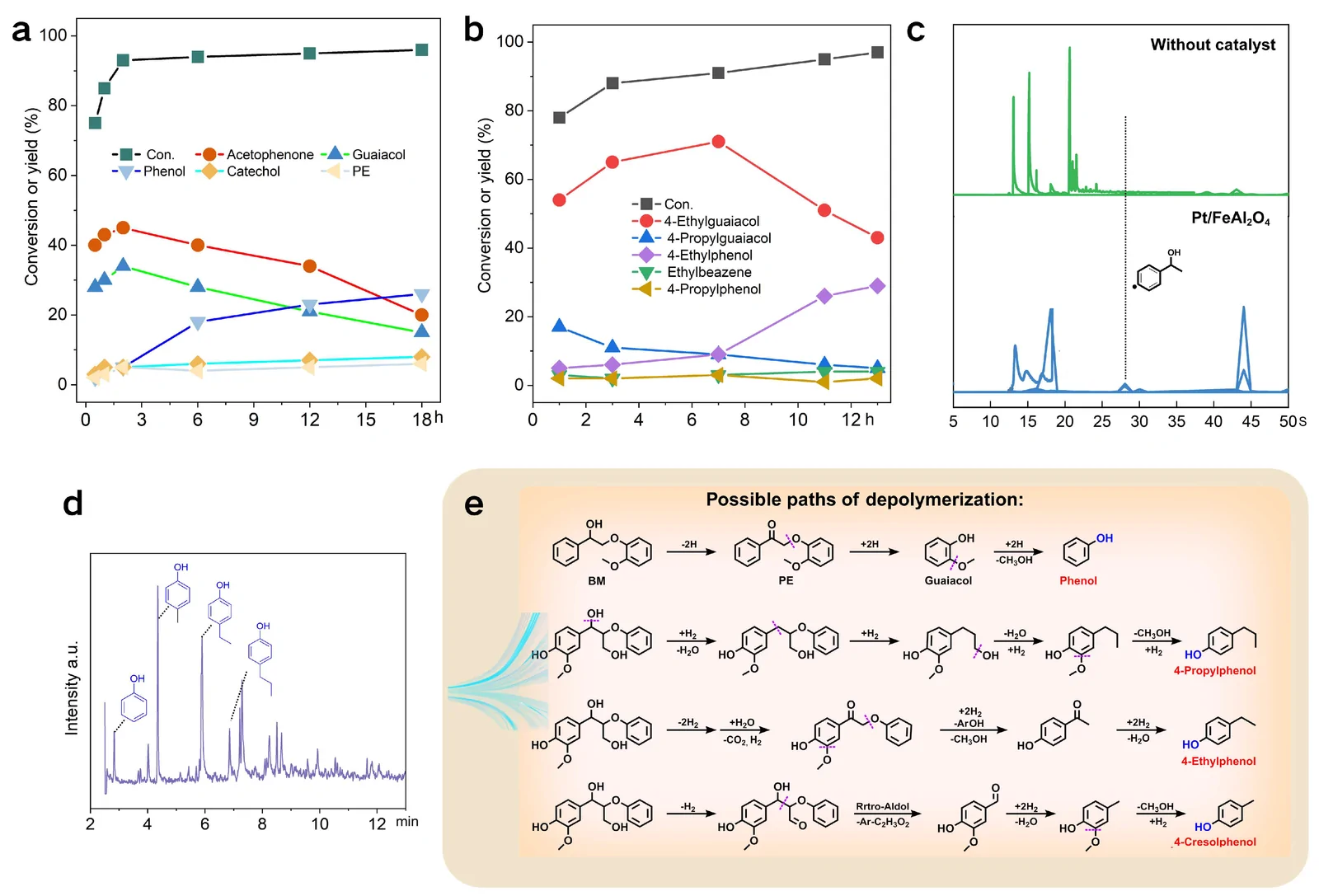
Mechanistic analysis of lignin depolymerization. (**a**,**b**) Time-resolved reaction profiles of BM and PG-OH. (**c**) Py-GC-MS profiles of 1-(4-methoxyphenyl)ethanol (MP-ol). (**d**) Product distribution during lignin depolymerization. (**e**) Proposed reaction pathways to p-substituted phenols.

**Figure 6:**
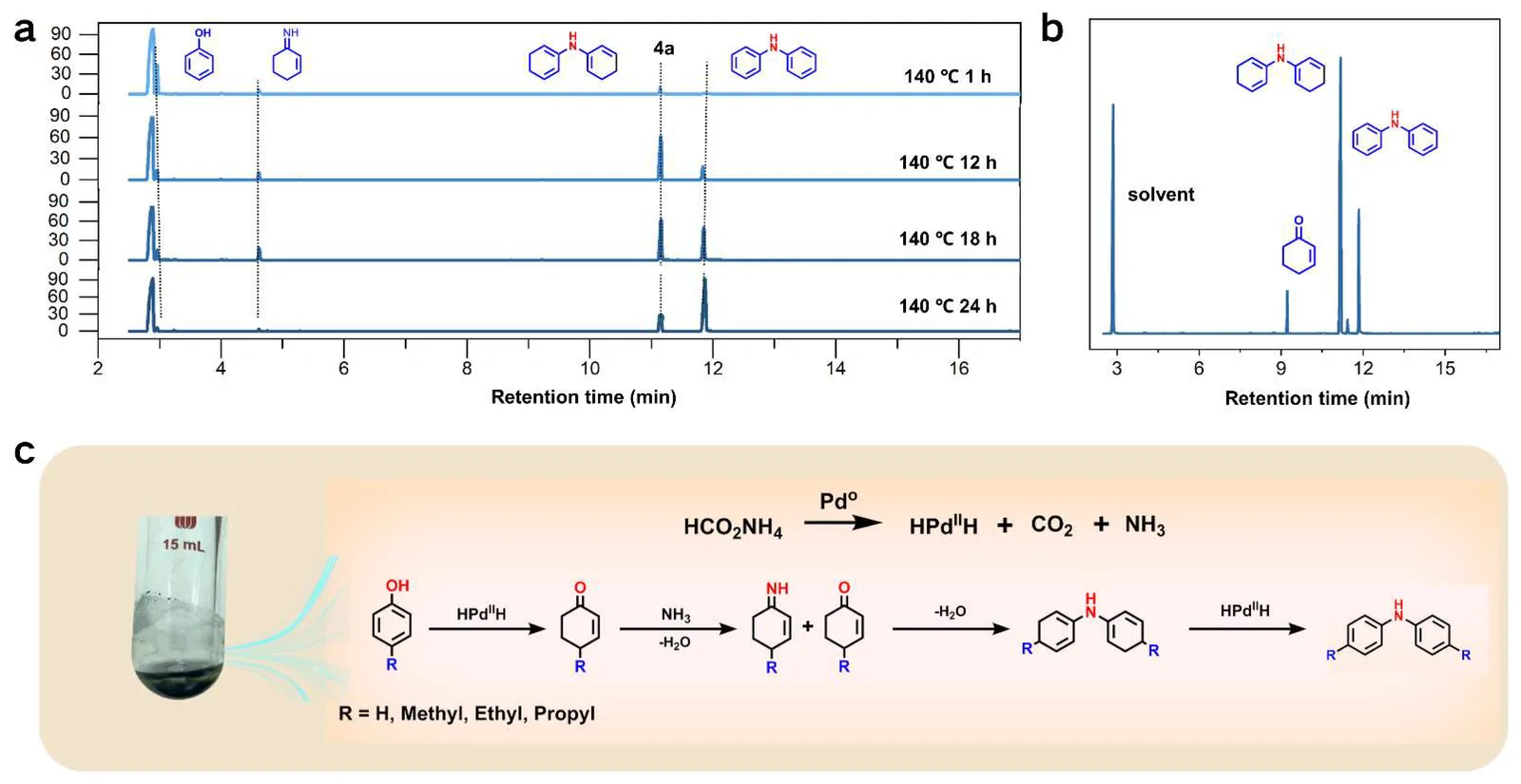
Mechanistic analysis of diphenylamine formation. (**a**) Time-resolved monitoring of the phenol reaction. (**b**) GC-MS analysis of the cyclohexenone intermediate in the amination reaction. (**c**) Proposed pathway to diphenylamine compounds.

**Figure 7:**
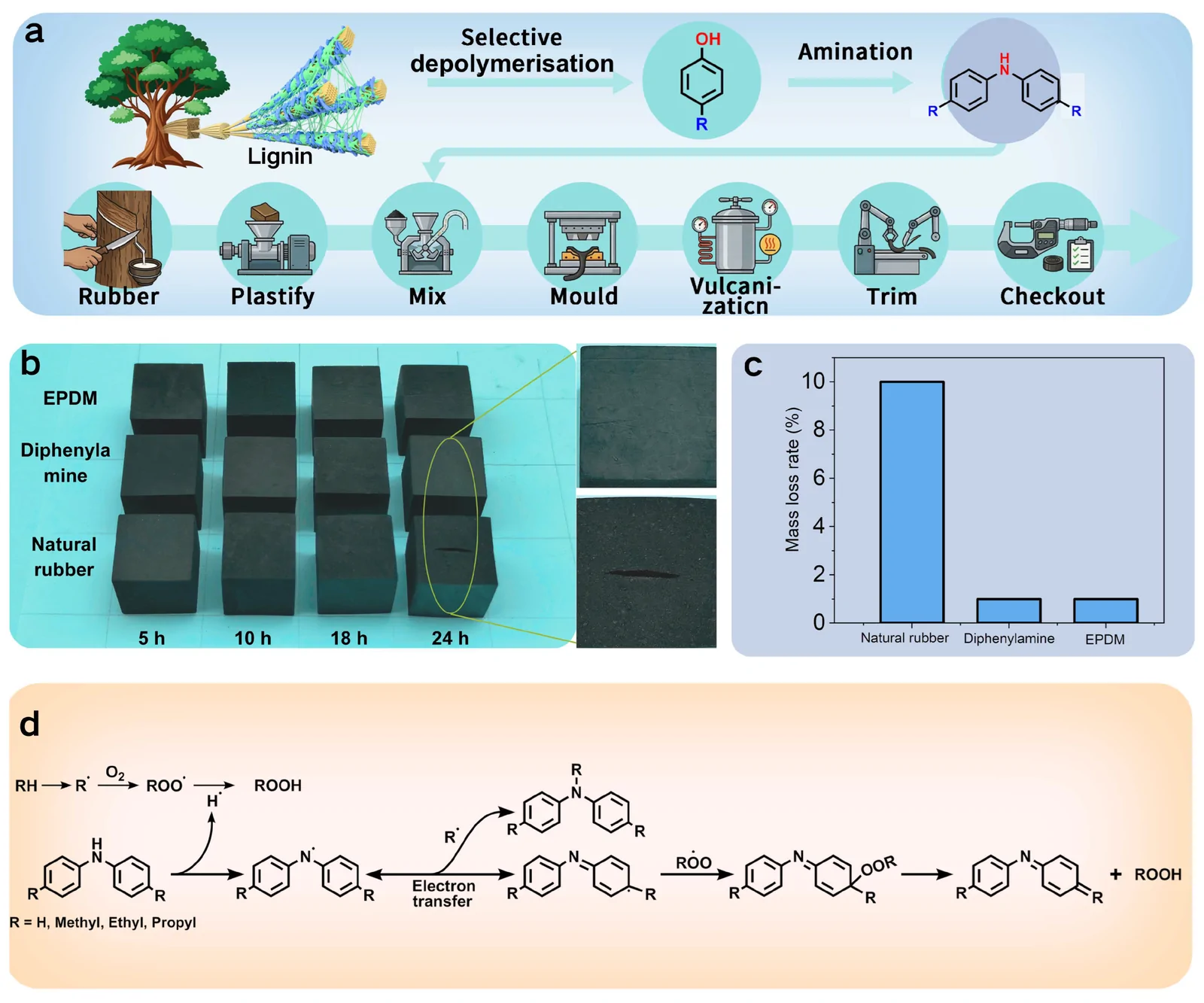
Antioxidant performance of lignin-derived diphenylamine compounds in natural rubber. (**a**) Schematic illustration of natural rubber containing diphenylamine compounds. (**b**) Photographs after thermal aging. (**c**) Mass loss after aging at 140 °C for 24 h. (**d**) Proposed antioxidant mechanism in the rubber matrix.

## Data Availability

The original contributions presented in this study are included in the article/Supplementary Materials. Further inquiries can be directed to the corresponding authors.

## Supplementary Materials

The following supporting information can be downloaded at: https://www.mdpi.com/article/10.3390/polym18202526/s1, Figure S1: A flowchart of the overall study process; Figure S2: XRD peak-fitting pattern; Figure S3: The particle size distribution of the catalyst; Figure S4: Adsorption–desorption isotherm of the catalyst; Figure S5: XPS spectrum of the catalyst; Figure S6: Effect of catalyst loading on p-substituted phenols; Figure S7: GPC analysis of birch lignin and its reaction residue; Figure S8: Evaluation of the reusability of the catalyst; Figure S9: Variation in pore size of the catalyst before and after use; Figure S10: Elemental distribution of the catalyst after use; Figure S11: Effect of reaction time on the yield of diphenylamine; Figure S12: 1H and ^13^C NMR spectra of various diphenylamine compounds; Figure S13: 2D-HSQC NMR spectroscopy of lignin structural changes at different reaction times; Figure S14: Photograph of the rubber block after aging at 160 °C for 24 h; Figure S15: DSC and FT-IR spectra of rubber samples; Table S1: Monomer concentrations in various lignins; Table S2: Textural parameters of catalyst; Table S3: Detailed vulcanization procedure; Table S4: Chemical formulas and reagents; Table S5: Abbreviations, acronyms, and analytical terms.
