# Supplementary material for: Lignin-Derived Diphenylamines for Thermo-Oxidative Stabilization of Natural Rubber
Source: Polymers (Basel). 2026 Oct 15;18(20):2526. doi: 10.3390/polym18202526 (PMC13709165; doi:10.3390/polym18202526)
Supplement: Supplementary file 1 [file polymers-18-02526-s001.zip › polymers-4462080-supplementary.pdf]

Supporting Information

Lignin-Derived Diphenylamines for Thermo-Oxidative Stabilization of Natural Rubber

Zhongwei Wang [1,2,†], Zhaozhuo Yu [1,†], Zhu Liu [1,2], Qinghan Zeng [1], Mei Luo [1], Yipeng Tang [1], Ning Ji [1,*], Wulin Yang [1] and Shoulu Yang [1,*]

1 Guizhou Academy of Forestry, Guiyang 550005, China; zhongweiwang202204@163.com (Z.W.)

2 College of Materials Science and Engineering, Central South University of Forestry and Technology, Changsha 410000, China

* Correspondence: jining420@163.com (N.J.); yangshoulucsuft@163.com (S.Y.)

† These authors contributed equally to this work.

Table of contents:

Figure S1. A flowchart of the overall study process.

Figure S2. XRD peak-fitting pattern.

Figure S3. The particle size distribution of the catalyst.

Figure S4. Adsorption–desorption isotherm of the catalyst.

Figure S5. XPS spectrum of the catalyst.

Figure S6 Effect of catalyst loading on p-substituted phenols.

Figure S7. GPC analysis of birch lignin and its reaction residue.

Figure S8. Evaluation of the reusability of the catalyst.

Figure S9 Variation in pore size of the catalyst before and after use.

Figure S10 Elemental distribution of the catalyst after use.

Figure S11. Effect of reaction time on the yield of diphenylamine.

Figure S12. ^{1}H and ^{13}C NMR spectra of various diphenylamine compounds.

Figure S13. 2D-HSQC NMR spectroscopy of lignin structural changes at different reaction times.

Figure S14. Photograph of the rubber block after aging at 160 °C for 24 hours.

Figure S15. DSC and FT-IR spectra of rubber samples.

Table S1. Monomer concentrations in various lignins.

Table S2. Textural parameters of catalyst.

Table S3. Detailed vulcanization procedure

Table S4. Chemical formulas and reagents

Table S5. Abbreviations, acronyms, and analytical terms

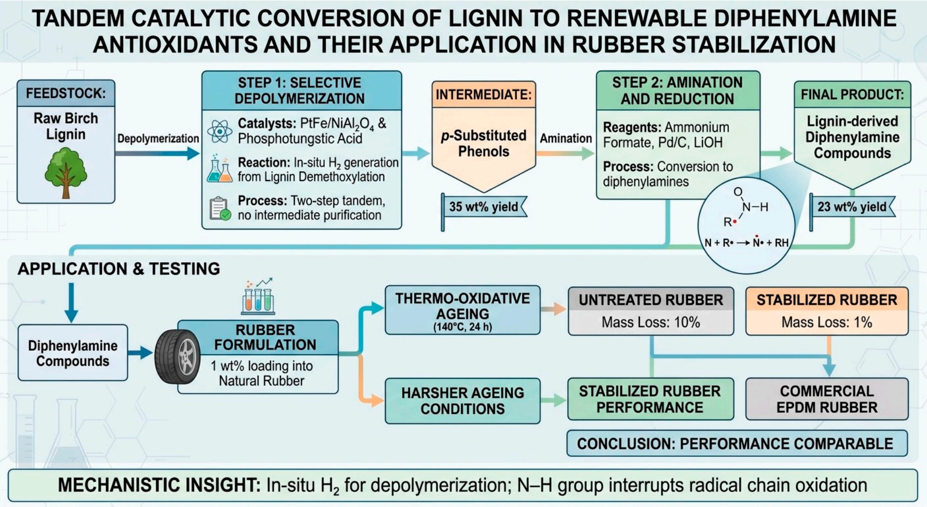

Figure S1. A flowchart of the overall study process.

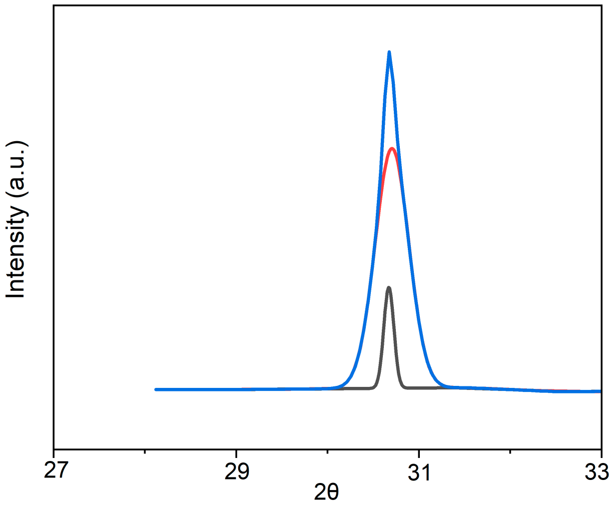

Figure S2. XRD peak-fitting pattern.

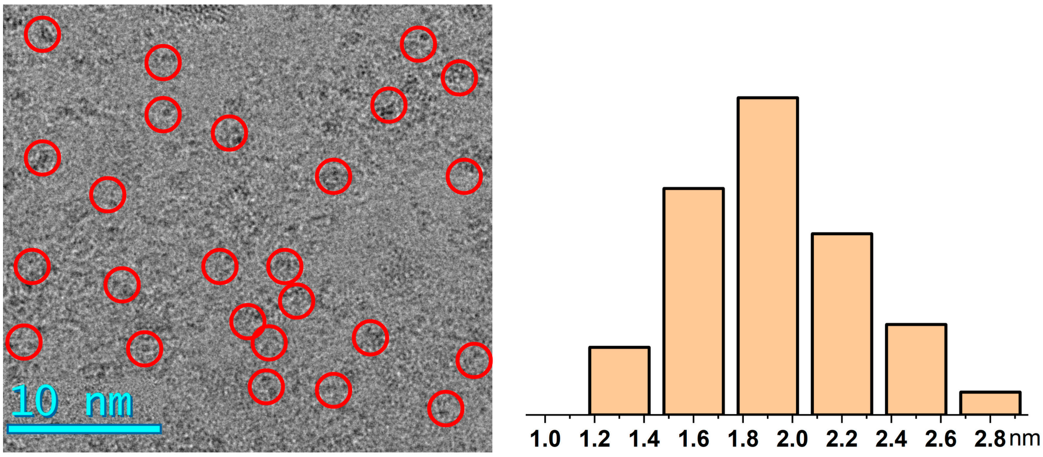

Figure S3. The particle size distribution of the catalyst.

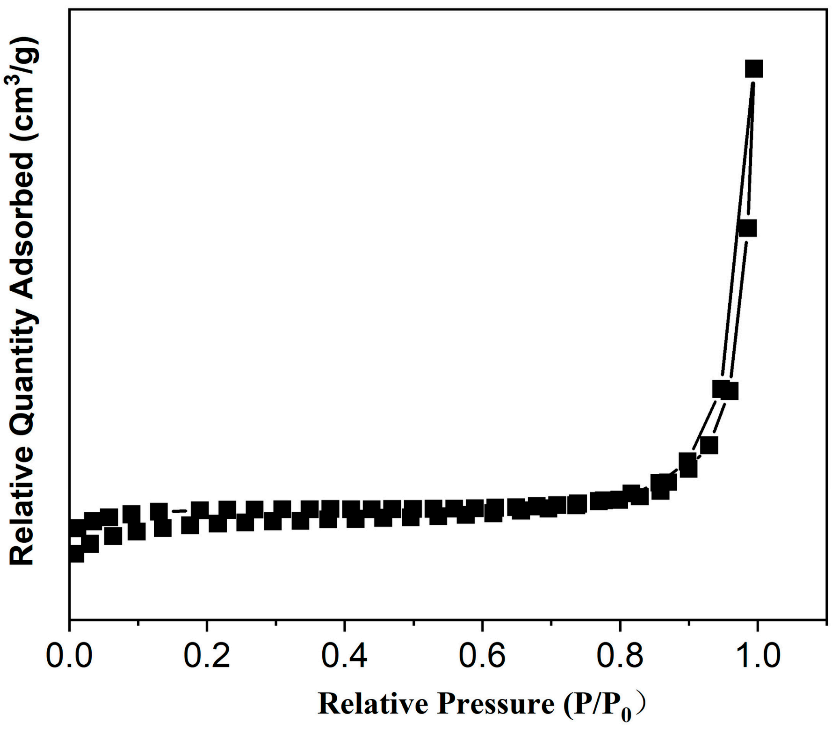

Figure S4. Adsorption–desorption isotherm of the catalyst.

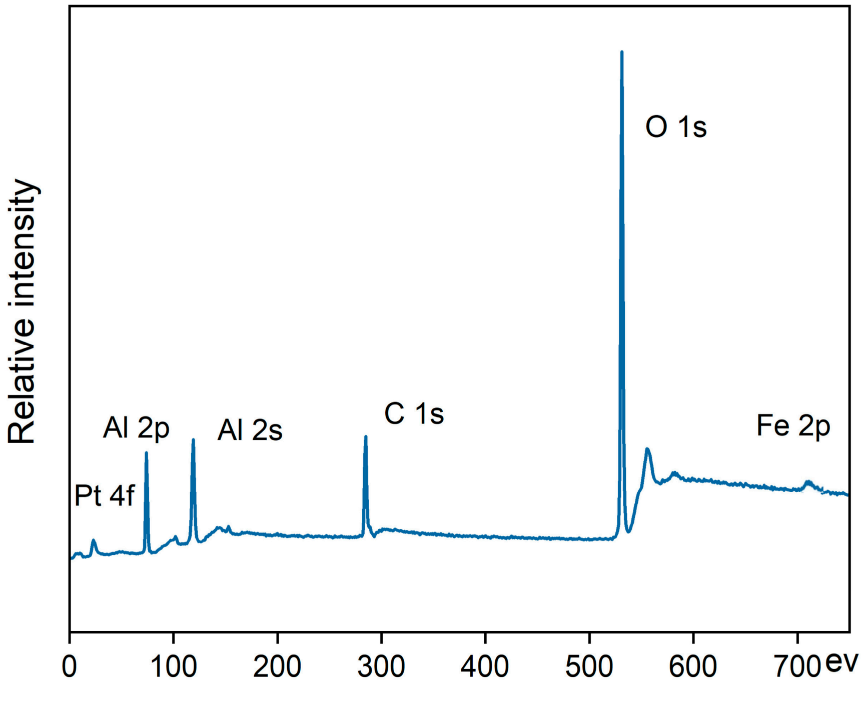

Figure S5. XPS spectrum of the catalyst.

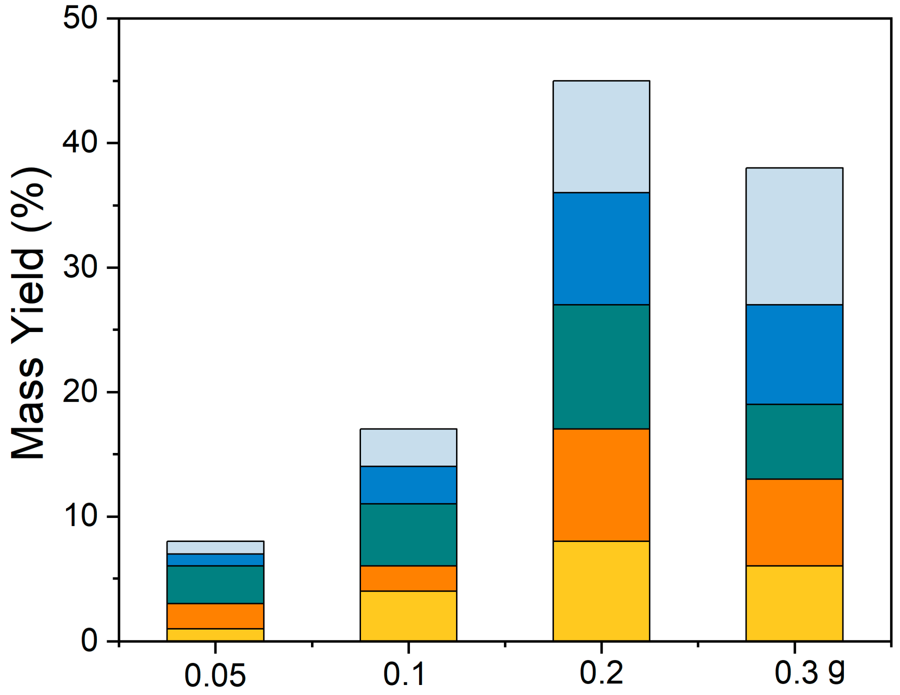

Figure S6. Effect of catalyst loading on p-substituted phenols. The standard deviation of the yield of p-substituted phenols is ±1%.

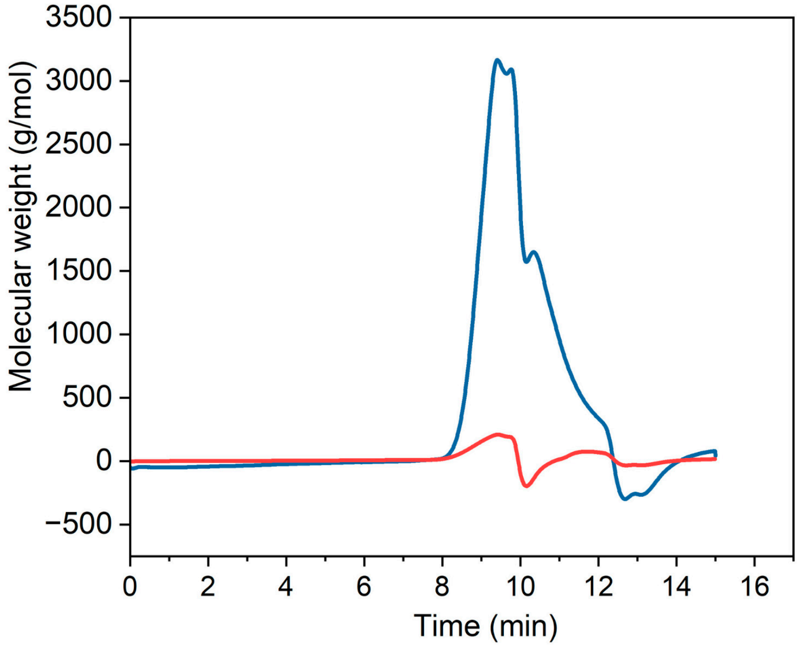

Figure S7. GPC analysis of birch lignin and its reaction residue.

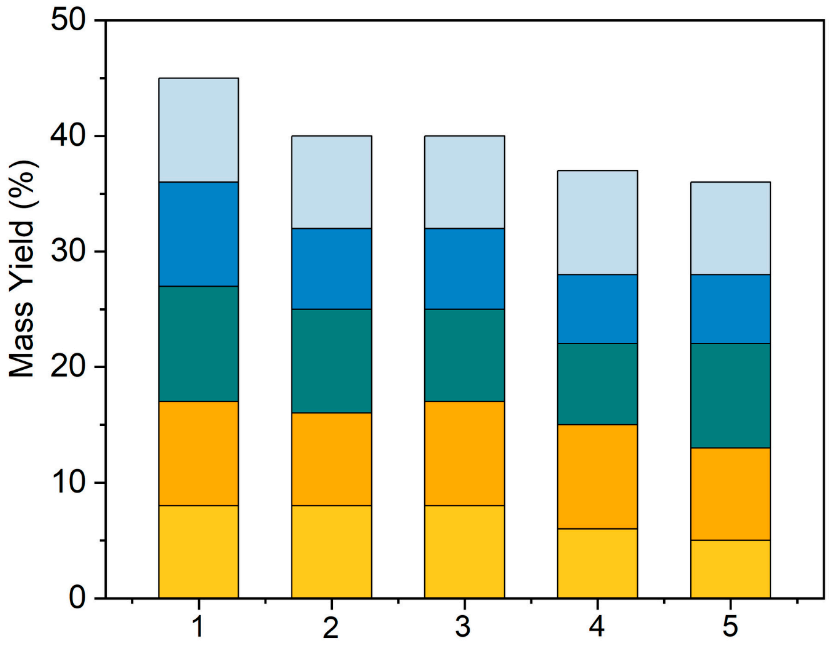

Figure S8. Evaluation of the reusability of the catalyst.

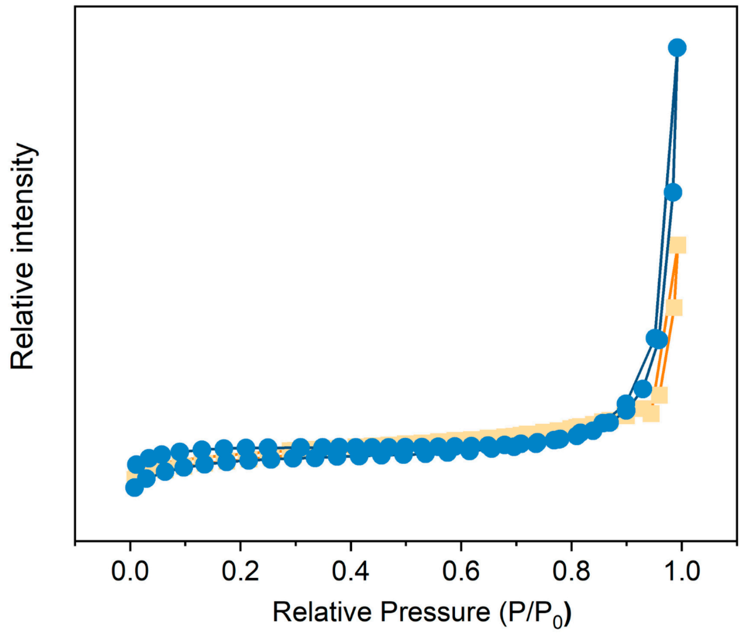

Figure S9 Variation in pore size of the catalyst before and after use.

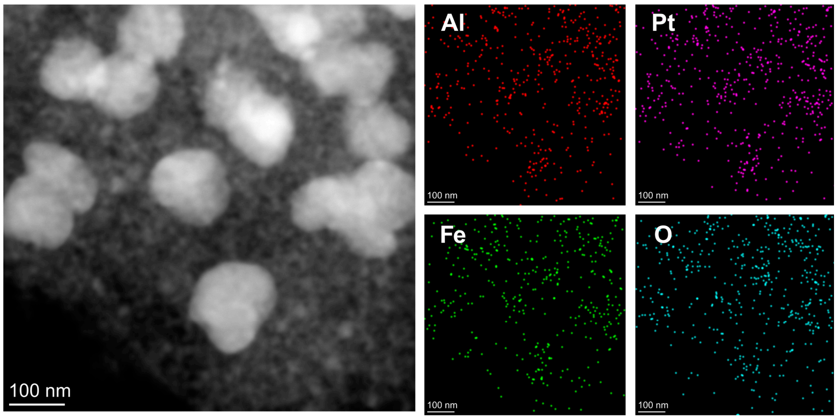

Figure S10 Elemental distribution of the catalyst after use.

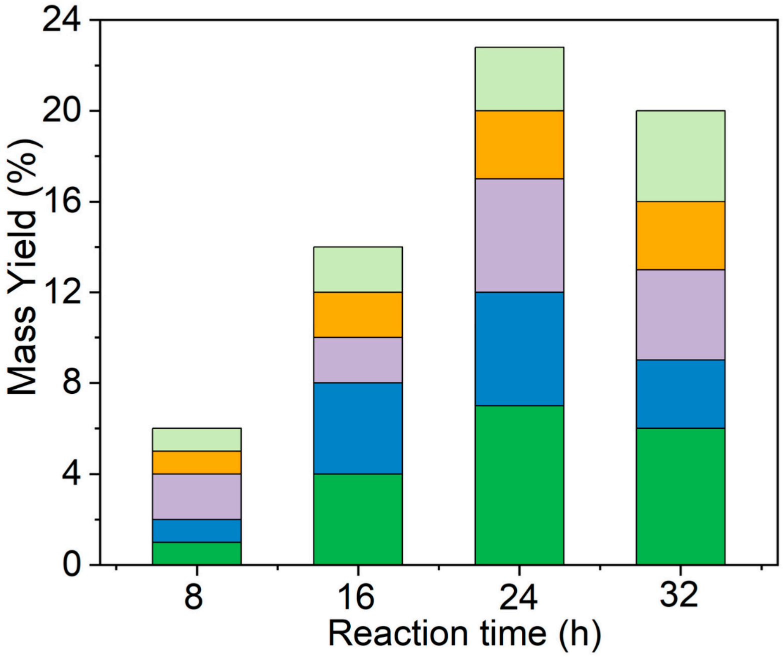

Figure S11. Effect of reaction time on the yield of diphenylamine. The standard deviation of the yield of diphenylamine compounds is ±0.5%.

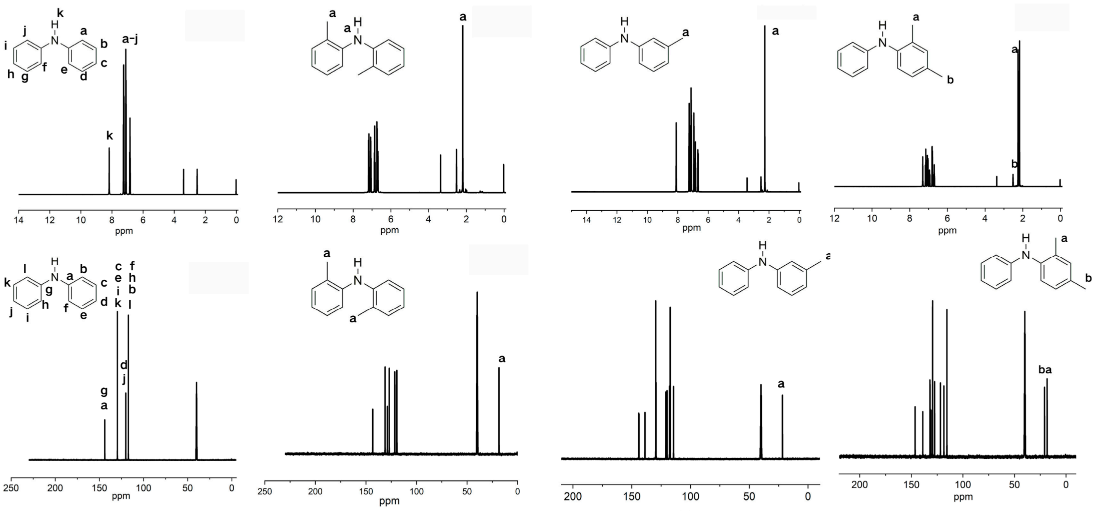

Figure S12. ^{1}H and ^{13}C NMR spectra of various diphenylamine compounds.

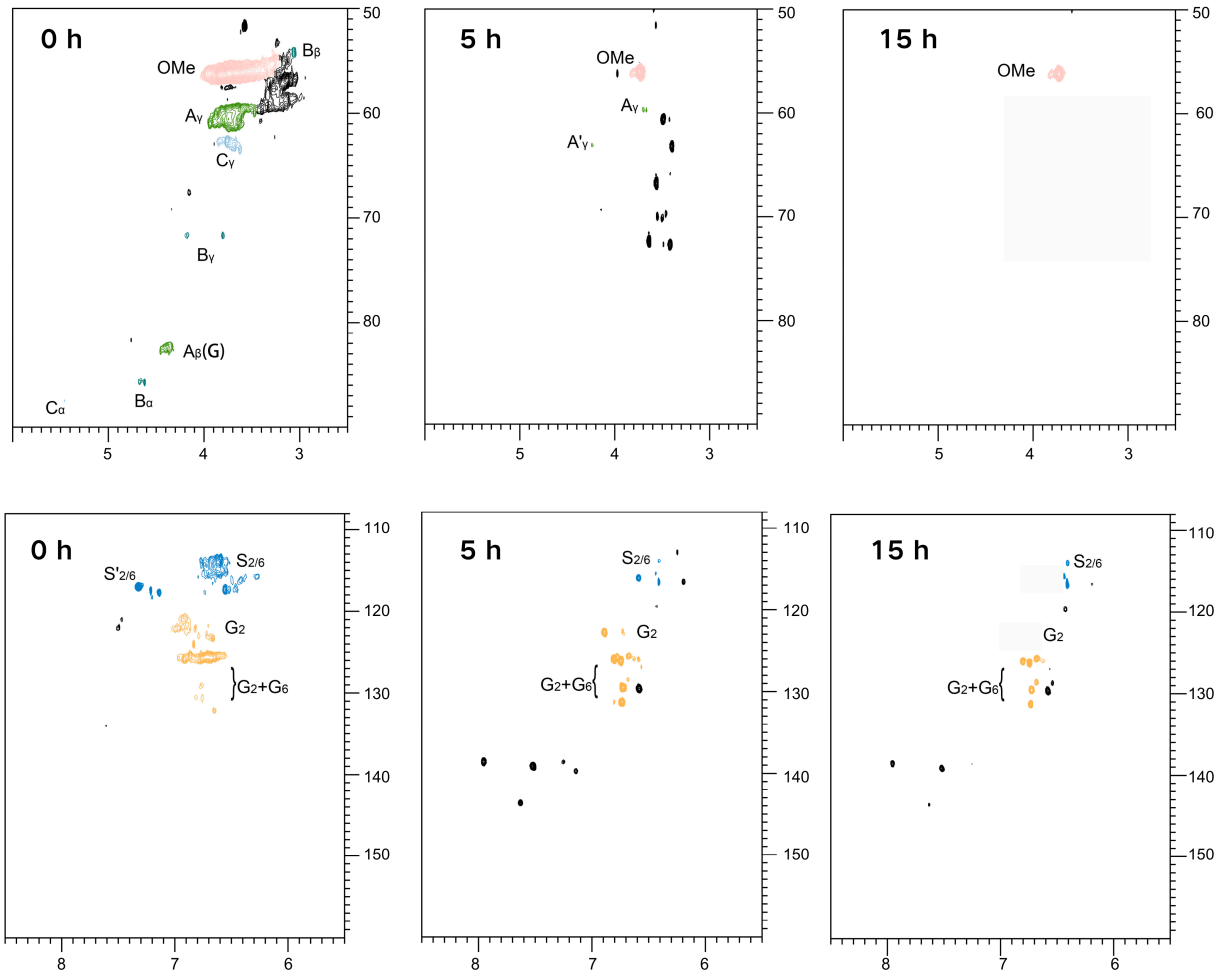

Figure S13. 2D-HSQC NMR spectroscopy of lignin structural changes at different reaction times.

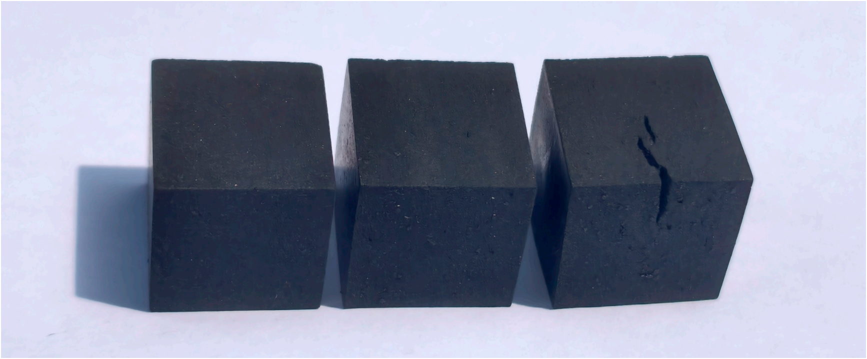

Figure S14. Photograph of the rubber block after aging at 160 °C for 24 hours.

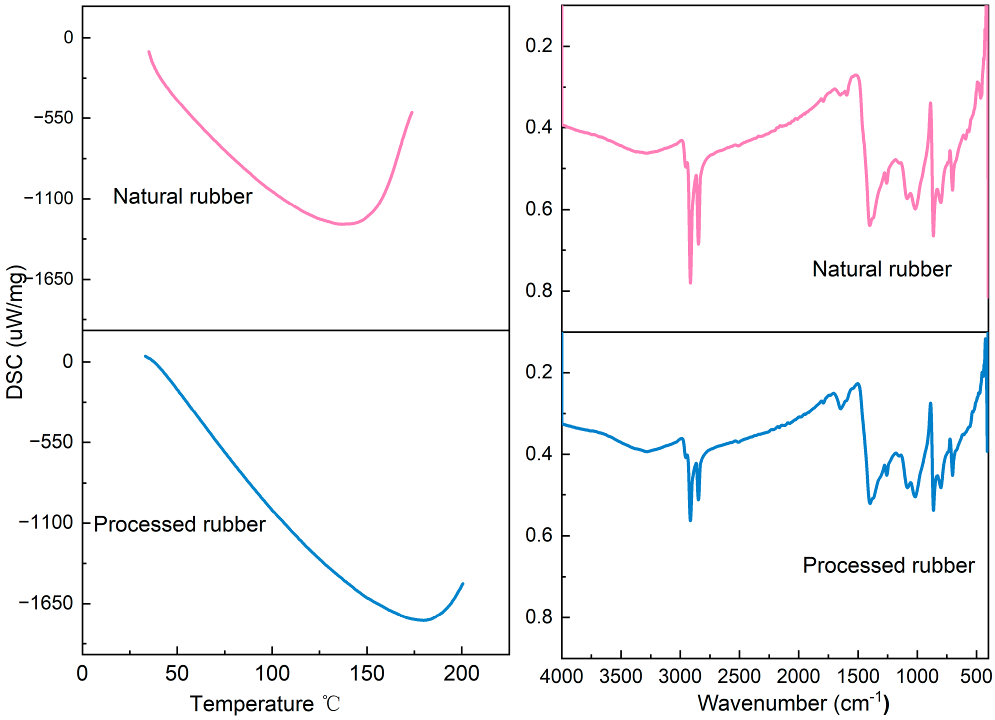

Figure S15. DSC and FT-IR spectra of rubber samples.

Table S1. Monomer concentrations in various lignins.

Entry	Lignin	S (/μmol·g-1)	G (/μmol·g-1)	H (μmol·g-1)	-OCH_3 (μmol·g-1)
1	Birch	2200	238	53	4690
2	Poplar	560	480	21	1801
3	Camphor	945	470	70	2478
4	Beech	1120	500	45	3268

Table S2. Textural parameters of catalyst.

Entry	Sample	BET surface area (m^2g^{-1})	Average Pore size (nm)	BJH pore volume ($\times 10^3$, cm^3g^{-1})
1	$PtFe/NiAl_2O_4$ flessh	556.1	3.5	1.43
2	$PtFe/NiAl_2O_4$ used	403.5	2.7	1.12

Table S3. Detailed vulcanization procedure.

Step	Operation	Key Parameters & Details
1	Based on the cavity volume of the 10 cm × 10 cm × 10 cm mold, weigh out an amount of the mixed compound that is slightly more than the final product weight.	• Mold cavity: 10 × 10 × 10 cm<br>• Compound weight: slightly over target product weight (to ensure complete filling and proper flash)
2	Preheat the mold on the heated platen of the plate vulcanization press.	• Temperature: 150 °C<br>• Duration: 30 minutes
3	Place the prepared compound blank into the preheated mold cavity, then close the mold.	• Ensure the compound fills the cavity evenly<br>• Close the mold promptly to prevent scorching
4	Raise the pressure to the set value. Immediately after reaching this pressure, perform a venting operation by quickly releasing the pressure and then reapplying it, so as to expel any trapped air from the compound.	• Pressure set value: 15 MPa<br>• Venting: quick release, reapply immediately
5	Maintain the pressure and temperature for the designated time to complete the vulcanization.	• Temperature: 150 °C<br>• Pressure: 15 MPa<br>• Duration: 20 minutes
6	After the vulcanization time has elapsed, release the pressure and open the press. Remove the mold from the platens.	• Depressurize completely before opening
7	Allow the mold to cool to room temperature, then open it and take out the finished product.	• Cooling: ambient / room temperature<br>• Demold carefully to avoid damaging the product

Table S4. Chemical formulas and reagents.

Formula /Notation	Full Name / Description	Context / Role in Article
$NiAl_2O_4$	Nickel aluminate	Spinel catalyst support
$PtFe/NiAl_2O_4$	Platinum–iron supported on nickel aluminate	Bimetallic catalyst for lignin depolymerization
$Ni(NO_3)_2$ $6H_2O$	Nickel(II) nitrate hexahydrate	Metal precursor for spinel synthesis
$Al(NO_3)_3$ $9H_2O$	Aluminum nitrate nonahydrate	Metal precursor for spinel synthesis
H_2PtCl_6	Chloroplatinic acid	Platinum precursor solution (10%)
$Fe(NO_3)_3$	ron(III) nitrate	Iron precursor solution (10%)
Pd/C	Palladium on activated carbon	Heterogeneous catalyst for amination step
LiOH	Lithium hydroxide	Basic promoter for amination

		polycondensation
HPW	Phosphotungstic acid	Protonic acid co-catalyst
N_2	Nitrogen gas	Inert atmosphere for calcination and reaction
H_2/Ar	Hydrogen in argon	Gas mixture (10%) for catalyst reduction
MgO	Magnesium oxide	Desiccant/basic additive evaluated in screening
CaO	Calcium oxide	Desiccant/basic additive evaluated in screening
KOH	Potassium hydroxide	Basic promoter evaluated in screening
ZnO	Zinc oxide	Rubber compounding agent
HCOONH4	Ammonium formate	Nitrogen donor and hydrogen source for amination polycondensation
S	Elemental sulfur	Vulcanizing agent for natural rubber matrix
C	Carbon black	Rubber compounding reinforcing filler

Table S5. Abbreviations, acronyms, and analytical terms.

Abbreviation	Full Term / Meaning	Context / Role in Article
AR	Analytical Reagent	Purity grade of chemical reagents
BET	Brunauer–Emmett–Teller	Surface area determination method via physisorption
BM	(2-Methoxyphenoxy)methyl]benzyl alcohol	Model dimer compound containing linkage
DDA	Diphenylamine	Commercial antioxidant benchmark / target class
2D-HSQC NMR	Two-Dimensional Heteronuclear Single Quantum Coherence Nuclear Magnetic Resonance	Characterization technique used to track lignin linkage degradation
EDS	Energy-Dispersive X-ray Spectroscopy	Elemental mapping technique used in STEM
EPDM	Ethylene Propylene Diene Monomer	Commercial synthetic rubber used as performance benchmark
G	Guaiacyl	Monomer unit structure in native lignin
GC-FID	Gas Chromatography–Flame Ionization Detector	Instrument used for product quantification
GC-MS	Gas Chromatography–Mass Spectrometry	Instrument used for qualitative compound identification
GPC	Gel Permeation Chromatography	Technique used to measure lignin molecular weight distribution
H	p-Hydroxyphenyl	Monomer unit structure in native lignin
HAADF-STEM	High-Angle Annular Dark-Field Scanning Transmission Electron Microscopy	High-resolution imaging technique for catalytic nanoparticle visualization
HPLC-UV	High-Performance Liquid Chromatography with Ultraviolet Detection	Instrument used for diphenylamine quantification
H_2-TPR	Hydrogen Temperature-Programmed Reduction	Analysis used to determine catalyst reducibility and metal interactions
ICP-OES	Inductively Coupled Plasma Optical Emission Spectrometry	Elemental analysis technique used to quantify Pt and Fe loading
Mn	Number-average molecular weight	Polymer chain length metric measured by GPC
MP-ol	1-(4-Methoxyphenyl)ethanol	Lignin model compound used in Py-GC-MS demethoxylation study
ODA	Octylated Diphenylamine	Commercial amine antioxidant variant
PG-OH	4-(3-Hydroxypropyl)-2-methoxyphenol	Lignin-derived monomer model compound
phr	Parts per hundred rubber	Unit for compounding formulation proportions
PTFE	Polytetrafluoroethylene	Material lining of the stainless-steel autoclave
Py-GC-MS	Pyrolysis Gas Chromatography–Mass Spectrometry	In situ monitoring technique for thermal transformation pathways
S	Syringyl	Monomer unit structure in native lignin
SAED	Selected Area Electron Diffraction	Technique used to verify crystalline alloy phases
STEM	Scanning Transmission Electron Microscopy	Microscopic structural analysis
TEM	Transmission Electron Microscopy	Microscopic structural and particle size analysis
TPSR	Temperature-Programmed Surface Reaction	Mechanistic study technique for tracing reaction intermediate evolution
XPS	X-ray Photoelectron Spectroscopy	Surface chemical state and binding energy analysis
XRD	X-ray Diffraction	Phase and crystalline structure characterization method
DSC	Differential Scanning Calorimetry	Thermal analysis of oxidation behavior in rubber

FT-IR	Fourier-Transform Infrared Spectroscopy	Characterization of carbonyl oxidation in rubber
